# The Success Level of Hypospadias Repair in Adults

**DOI:** 10.7759/cureus.9108

**Published:** 2020-07-10

**Authors:** Sami Ullah, Sundas Karimi, Haroon Sabir Khan, Umar Farooque, Omer Cheema, Priyanka Kumari, Komal Girdhari, Naresh Kumar, Fahad N Sheikh, Maleeha Ali Basham, Farah Yasmin, Rizwan Farooque

**Affiliations:** 1 Urology, Pakistan Navy Ship (PNS) Shifa Hospital, Karachi, PAK; 2 General Surgery, Combined Military Hospital, Karachi, PAK; 3 Neurology, Dow University of Health Sciences, Karachi, PAK; 4 Internal Medicine, Dow University of Health Sciences, Karachi, PAK; 5 Internal Medicine, Chandka Medical College, Larkana, PAK; 6 Internal Medicine, Civil Hospital, Sukkur, PAK; 7 Medicine, Dow University of Health Sciences, Karachi, PAK; 8 Pathology, Sahiwal Medical College, Sahiwal, PAK; 9 Cardiology, Dow University of Health Sciences, Karachi, PAK; 10 Internal Medicine, Sindh Medical College, Karachi, PAK

**Keywords:** hypospadias, hypospadias repair, tip urethroplasty, success level, adults, age, humans

## Abstract

Introduction

Hypospadias is the abnormal opening of the urethra at the undersurface of the penis. Hypospadias repair is performed in such patients to treat the condition. The success level of hypospadias repair in adults still needs to be established on a larger scale. Therefore, we conducted this study to document the success level of hypospadias repair in adults in our setting to add to the literature.

Materials and methods

This prospective study was conducted at a major metropolitan hospital in Karachi over a period of six months. A total of 75 male patients aged between 20-50 years and diagnosed with hypospadias of any level with or without mild to moderate chordee were included. Demographic features such as age and the duration of hypospadias were noted. Hypospadias repair using a tubularized incised plate (TIP) urethroplasty technique was performed, and the patients were observed for three days in the ward and for three months in the outpatient department for any complications. The procedure was considered a success if there were no complications and no need for a second surgery; we also took into account patient satisfaction with the procedure to determine the success level. The mean and standard deviation were calculated for patient age and the duration of hypospadias. Frequency and percentages were calculated for distribution of patient age, distribution of the duration of hypospadias, and the success of hypospadias repair. The correlation of patient age and the duration of hypospadias with the success of hypospadias repair was also determined by applying the Chi-square test, and a p-value of ≤0.05 was considered to be statistically significant.

Results

The mean age and the duration of hypospadias for the study population were the same at 31.43 ± 8.47 years. Distribution of patient age and the duration of hypospadias was also the same with 51 (68%) patients of ≤35 years of age and 24 (32%) patients of >35 years of age. Hypospadias repair was successful in 52 (69.33%) patients but unsuccessful in 23 (30.66%). The stratification of patient age and the duration of hypospadias with the success of hypospadias repair showed a significant inverse relationship (p = 0.017). The data relating to patient age and the duration of hypospadias showed the same values as hypospadias is a birth defect.

Conclusion

Adult patients undergoing primary hypospadias repair generally show good outcomes. However, the chances of favorable outcomes gradually decrease with age. Hence, patients should be encouraged to undergo the procedure as early in their lives as possible. Patients who are middle-to-old aged should especially be counseled about the relatively higher risk of unsuccessful procedures. Further analysis is needed to confirm the validity of these findings.

## Introduction

Hypospadias is a condition characterized by the congenital opening of the urethra on the undersurface of the penis. It occurs in one in every 300 boys and is the second most common congenital defect in boys [1]. It is also associated with underdeveloped foreskin and chordee [2]. The extremes of maternal age (>35 years and <18 years), maternal consumption of alcohol and drugs, and infection during pregnancy are associated with an increased risk of hypospadias. Its risk is also higher among children of farmers [3]. The actual pathogenesis of the association between hypospadias and the aforementioned risk factors is still unclear [4].

Hypospadias becomes apparent during circumcision or after foreskin retraction during late childhood. Circumcision does not affect the hypospadias repair success levels in children with fully developed foreskin and absence of chordee [5]. Hypospadias repair is the preferred treatment option, and it is successful in most of the patients. A few patients may need multiple procedures and can develop scarring, curvature, urethral fistulas, or strictures [6]. The outcomes of hypospadias repair may or may not be affected by the age at which the repair is done, and the patients usually spend one night in the hospital post-surgery [7,8,9].

The current literature shows that hypospadias repair is successful in 88%-95% of adult patients [8,9]. This study was conducted to determine the success level of hypospadias repair in adults in our setting. We believe our findings will be a valuable addition to the already available data.

## Materials and methods

Study design and sampling

This prospective study was conducted at the Jinnah Postgraduate Medical Center, Karachi, from December 10, 2018, to June 10, 2019 (six months). The non-probability consecutive sampling technique was applied. The sample size of 75 patients was calculated by using the Epi Info 7 and keeping the confidence level at 95%, the margin of error at 10%, and the expected percentage of success at 76.5% [8]. The inclusion criteria were as follows: male patients between the ages 20-50 years with a diagnosis of hypospadias of any level with or without mild to moderate chordee. Patients with a previous unsuccessful repair within the last 12 months, a history of infection after the previous repair, diabetes mellitus, severe chordee, and erectile dysfunction were excluded from the study.

Data collection

All patients meeting the inclusion criteria were enrolled in this study. Patients were provided information about the study, and informed consent was obtained. Demographic factors such as patient age and the duration of hypospadias were collected. Hypospadias repair using a tubularized incised plate (TIP) urethroplasty technique was performed on all patients by a single surgical team under spinal anesthesia. The patients were kept under observation and their wound was examined for three days. After discharge, they were followed up in the outpatient department on a monthly basis for three months and were encouraged to approach the outpatient department in case of any complication. The repair was labeled successful if no complication occurred or a redo surgery was not required for three months; we also took into account the patient satisfaction with the procedure in determining the success level. All the data was recorded on a proforma.

Data analysis

Data was entered and analyzed on SPSS Statistics version 20 (IBM, Armonk, NY). The mean and standard deviation were calculated for patient age and the duration of hypospadias. Frequency and percentages were calculated for distribution of age, distribution of the duration of hypospadias, and the success of hypospadias repair. Stratification was performed to see the effects of the patient age and the duration of hypospadias on the success of hypospadias repair; the Chi-square test was applied and a p-value of ≤ 0.05 was considered significant.

## Results

The mean age and the duration of hypospadias for the patients were 31.43 years with a standard deviation of 8.47 years, as shown in Table 1.

**Table 1:** Analysis of patient age and duration of hypospadias

	Minimum	Maximum	Mean	Standard deviation
Age and duration of hypospadias (years)	20	50	31.43	8.47

**Figure 1:**
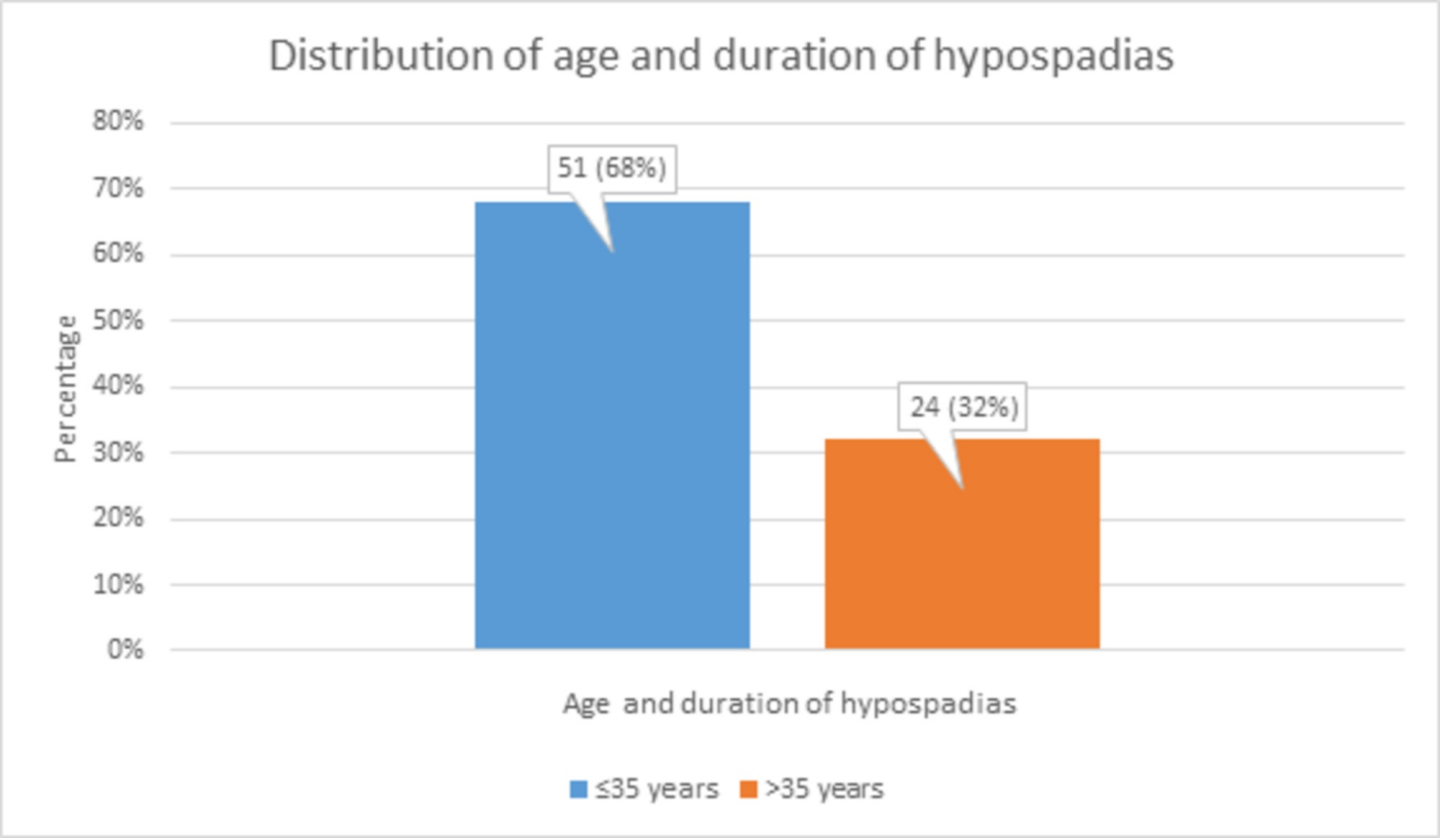
Distribution of patient age and duration of hypospadias

The distribution of patient age and the duration of hypospadias showed that there were 51 (68%) patients of ≤35 years of age and 24 (32%) patients of >35 years in age, as shown in Figure 1.

**Figure 2:**
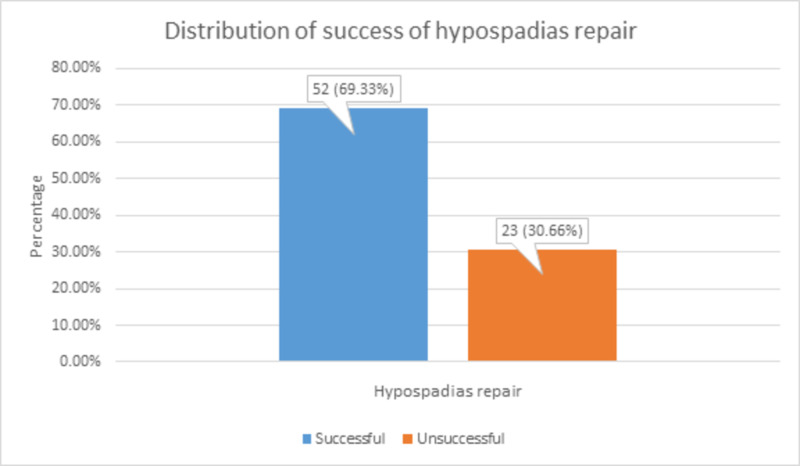
Distribution of success of hypospadias repair

The expected outcome of the study, i.e., the success of hypospadias repair, was found in 52 (69.33%) patients, while 23 (30.66%) repairs were unsuccessful, as shown in Figure 2.

The stratification of patient age and the duration of hypospadias with the success of hypospadias repair showed a significant inverse relationship with a p-value of 0.017, as shown in Tables 2.

**Table 2:** Stratification of patient age and duration of hypospadias with the success of hypospadias repair

Patient age and duration of hypospadias (years)	Success of hypospadias repair		Total, n (%)	P-value
	Yes, n (%)	No, n (%)		
≤35	40 (53.33%)	11 (14.66%)	51 (68%)	0.017
>35	12 (16%)	12 (16%)	24 (32%)	
Total	52 (69.33%)	23 (30.66%)	75 (100%)	

The data relating to patient age and the duration of hypospadias showed the same values because hypospadias is a congenital disorder.

## Discussion

This study showed a success level of 69.33% in hypospadias repair. While it was a pretty good outcome, it was a little lower than the levels described in the previously published literature [8,9]. At the same time, it also showed that the success rate of hypospadias repair decreases with increasing age and the duration of hypospadias.

Adayener et al. found that the success level of hypospadias repair in patients with a previous history of procedures was 76.5%, and it was 91.3% in patients with no previous history of procedures. The overall success level was found to be 88%. They also found that the success level decreased with age, especially in patients with a previous history of interventions [8]. AlTaweel et al. found that the success level of primary hypospadias repair was 71% while that of secondary hypospadias repair was 55%; the overall success level of hypospadias repair was 95%. They also found that the overall complication level was 60% [9].

The objective of hypospadias repair is to obtain a straight phallus with normal urination from the tip. After the repair, the assessment of its results can be obtained by Hypospadias Objective Scoring Evaluation (HOSE) and uroflowmetry. Other signs indicating the success of surgery include correction of chordee, a normal meatus, and a smooth straight urinary flow. The most common complication of hypospadias repair is the formation of fistula [10,11].

According to numerous studies, the most effective hypospadias repair technique is TIP urethroplasty, with a complication rate of only 6-16% [11,12]. Baskin et al. found that mild to moderate chordee can be easily corrected with TIP urethroplasty by skin mobilization and dorsal plication [13]. However, it has limited benefit in cases of severe chordee. The most commonly faced complication is meatal stenosis, which occurs due to excessive closure of distal meatus, but sequential graduation of new urethra or dilation can easily eradicate this problem [14,15].

The level of hypospadias and the presence or absence of chordee determine the success level of hypospadias repair. Mild chordee can be corrected by dorsal plication; however, severe chordee has to be corrected by a staged repair or tubularized flaps, but chances of fistula formation are much higher because of the numerous repairs performed at the same time [16,17]. Currently, to minimize the complications, numerous modifications are being made to the already existing procedures, which include the application of monofilament sutures, usage of special dressings like DuoDERM® dressing (ConvaTec International, Skillman, NJ) and silicone gel foam dressing, and enhancement of the size and blood supply of the phallus with androgen-containing creams or injections that aids in improved flap creation and healing of wounds [16,17].

## Conclusions

Primary hypospadias repair using the TIP urethroplasty technique generally leads to favorable outcomes in adult patients, but this success level declines with increasing age. Hence, it is better to perform hypospadias repair as early as possible in life, and adults undergoing hypospadias repair should be informed about the low level of success of hypospadias repair and high risk of complications as compared to procedures in children. Further studies on a larger scale and with longer periods of follow-up are necessary to determine the actual success level of hypospadias repair in adults and its relationship with patient age and the duration of hypospadias.
